# Supplementary material for: Compassionate Care: A Qualitative Exploration of Nurses’ Inner Resources in the Face of Burnout
Source: Nurs Rep. 2024 Jan 2;14(1):66–77. doi: 10.3390/nursrep14010006 (PMC10801579; doi:10.3390/nursrep14010006)
Supplement: Supplementary file 1 [file nursrep-14-00006-s001.zip › Document S1 Notice for student Communications Platform (Moodle).pdf]

Appendix

Appendix A.

Semi-structured Interview guide

Block 1: Introduction and 'Ice-breaker'
What aspects of the profession drew you to study nursing? / ¿Qué aspectos de la profesión te motivó a estudiar enfermería?
How would you define the role of nursing? / ¿Cómo definirías el trabajo de enfermería?
Considering your definition, do you do this on a daily basis? Why? / Con relación a tu definición, ¿consideras que es lo que haces en tu día a día? (¿Por qué sí o por qué no?
Have there been changes to your concept of nursing since you started/graduated? What are they? / ¿Ha habido algún cambio o cambios, respecto a tu concepto de enfermería, que haya cambiado desde que saliste de la facultad hasta en el momento actual? (¿porqué?)
How has the work environment changed / your attitude to patients / your care of patients? / ¿Ha cambiado el entorno? ¿tu actitud con los pacientes? ¿el cuidado a los pacientes?
**Block 2: Work Conditions and Professional Quality of Life**
What does a "normal" shift look like for you? / ¿Cómo es un día de trabajo "normal" para Ud.?
What Word/s define your working conditions?<br>What are the main challenges you face on a day to day basis? / ¿Cuáles son los principales retos a los que se enfrenta en el trabajo?
Do you consider that nurses are overworked on your unit? / ¿Considera que en su unidad hay sobrecarga de trabajo sobre las enfermeras?
If so, how does this affect you? / ¿En caso de que sea así, como le afecta?
Considering the work burden on your unit / Debido a la sobrecarga, o no…
- What challenges do you face in order to deliver health care? / ¿A qué retos se enfrenta para prestar los cuidados en salud?
- Are there aspects of care delivery that become more difficult? Hay algún aspecto en este acto de prestar cuidados que le cueste más de hacer?
- What aspecto of care do you most enjoy providing? / Hay algún aspecto en este acto de prestar cuidados que le gusta más realizar?
How would you define the "ideal" shift with regards to care delivery / Para Ud. cómo definiría un turno ideal con relación a la prestación de cuidados?
Do you feel respected / cared for in your work environment? How so? / ¿Se siente respetado/a y cuidado/a en su entorno de trabajo? ¿Por qué sí o Por qué no?
What is the work-place atmosphere like? Examples? / ¿Considera que hay un buen ambiente laboral? ¿Por qué sí, por qué no?

Are there professional development opportunities? Is that important to you? / ¿Hay posibilidades de desarrollo profesional? ¿Es importante para usted?
How/Are you able to establish personal boundaries when delivering care? What are they? Why is it hard to place them? / ¿Establece algún tipo de límite personal a la hora de realizar los cuidados de enfermería?
Have you witnessed malpractice at work? How did it make you feel? What do you think the cause of it is? / ¿A nivel laboral, ¿ha visto *mala praxis*? ¿Cómo se sintió? ¿Cuál cree que es la causa?
In general, do you feel satisfied with your work and why? What factors attribute to this? / En general, ¿siente satisfacción con el trabajo? ¿Por qué sí o Por qué no?
Block 3: Internal Resources (Cognitive Capacity, Affect, Spirituality, Morality, Self-Awareness & Awareness of Others
COGNITIVE RESOURCES:
Do you consider yourself a consistent person when pursuing your goals? Do you think you are effective in solving your daily life problems? Do you consider yourself an optimistic person? Do you think these skills are important for your work and how does it make you feel? / ¿Se considera una persona constante cuando persigue sus objetivos? ¿Cree que es eficaz resolviendo sus problemas de la vida diaria? ¿Se considera una persona optimista? ¿Piensa que estas habilidades son importantes para su trabajo y cómo le hace sentir?
Have you ever felt stressed in your work environment?<br>Could you explain what you feel when you realise that you feel stressed (*use their word*), and how you deal with it?<br>Can you identify an aspect of your work that triggers stress?/ En algún momento ¿se ha sentido estresada/o en su entorno laboral? Me podría explicar que sintió para considerar que estaba estresada/do?, ¿Cómo lo gestiona?
In order to reduce your level of stress, what internal/external strategies do you use (e.g. relaxation techniques, exercise, smoking, etc.) Do you ask for external help? / Para disminuir el nivel de estrés, ¿utilizó alguna estrategia interna/externa para poder gestionarlo? (Me refiero a si puso en práctica técnicas de relajación, aumento el nivel de ejercicios, de fumar, etc. O bien a pesar de recurrir a alguna técnica tuvo que pedir ayuda externa?)
How have past experiences helped you prepare for the future? / ¿Las experiencias pasadas le han preparado para acciones futuras? ¿Cómo?
AFECTIVE RESOURCES:
Would you describe yourself as a cheerful, strong and enthusiastic person? Is this the same at work? Do you think that being like this has made your relationship with patients easier? Has this been positive for your working life? / En general, ¿se definiría como una persona alegre, fuerte y entusiasmada por las cosas? ¿Es así también en su trabajo? ¿Cree que ser así le ha facilitado la relación con los pacientes? ¿Y eso ha sido positivo para su vida laboral?

In what way does the way you feel affect the care you provide? /Según cómo se sienta Ud. ¿puede afectar a los cuidados que presta? ¿Por qué? ¿En qué sentido?
Can you tell when you are begining to get stressed at work, can you stop it? How can you tell? Can you stop it? / ¿Sabe si empieza a sentirse estresado en el trabajo, puede frenarlo o cambiarlo?
Have you ever experienced burnout at work? (If so) what did it feel like? /Alguna vez, ¿ha experimentado "el agotamiento" en su entorno laboral? ¿Cómo se sintió ante este "agotamiento"?
SPIRITUAL AND MORAL RESOURCES:
How would you define professional ethics?<br>Does religion have a role in your life? Does spirituality? Do these help you cope?<br>What does morality mean to you?<br>Do you think that ethical behaviour helps you to cope with your work?<br>Have you experienced situations that have created some kind of ethical stress /dilemma?<br>/ ¿Cómo definirías la ética profesional?¿Cree que este comportamiento ético le ayuda a sobrellevar su trabajo? ¿Ha vivido situaciones que le hayan creado algún tipo de estrés ético, algún tipo de dilema?
If I say "moral conflict", what comes to mind? Can you relate to that feeling, or give me an example?<br>Do you feel supported in these situations? By whom?<br>/ Si digo dilema/ conflicto moral, ¿qué le viene a la mente? ¿Puede relacionarlo/ dar un ejemplo? ¿Se siente apoyado, y de quién?
What is more important, ethics or being practical? Why? / ¿Es más importante la ética o los aspectos prácticos, cómo y por qué?
SELF-AWARENESS & OTHERS:
Do you consider yourself to be a person who lives in the here and now?<br>Do you feel connected to yourself?<br>Do you usually treat yourself with kindness?<br>Can you be patient with yourself and the things about yourself that you don't like? /<br>¿Se considera una persona que vive el aquí y ahora? ¿Se siente conectado consigo mismo? Habitualmente, ¿se trata a usted mismo con amabilidad? ¿Es paciente con usted y las cosas de sí mismo que no le gustan o agradan?
Do you have these feelings of connection, patience and awareness when you interact with others? Do you pay full attention when you talk and interact with others, accepting their opinions, even if they are different from yours?<br>/ Y estos sentimientos de conexión, paciencia y conciencia, ¿los tiene también cuando interactúa con los demás? ¿Presta atención plena cuando habla e interactúa con los demás, aceptando sus opiniones, aunque sean diferentes a la suya?

Do you think that an ability to be aware of oneself and others can be applied to your patients? What results does this have on your work? (Negative and or positive) / ¿Cree que esta capacidad de ser consciente de uno mismo y los otros, la aplica también con sus pacientes? ¿Qué resultados positivos en su trabajo tiene? ¿Cree que tiene aspectos o consecuencias negativas?
Has experience, allowed you to improve your management of problems (both work-related and emotional) How? /Con la experiencia, ¿ha podido mejorar la gestión o la forma de enfrentarse a los problemas laborales y/o emocionales derivados del trabajo? (¿Por qué sí o porque no, y cómo?)
How have your perceptions of nursing changed over time? ¿Han cambiado con el tiempo sus percepciones sobre la enfermería? En caso afirmativo, ¿cómo?
What has contributed to this change? / ¿Qué factores contribuyen al cambio?
**Self-Care:**
Does your free-time help to alleviate your feelings of work-induced stress, (or do you remain feeling anxious?) Could you talk me through it? / ¿Su tiempo libre o cuando no está trabajando, le ayuda a disminuir el estrés (ansiedad, nerviosismo…)?
What hobbies or interests do you have that help you to disconnect from work? (sport, nature, leisure) / ¿Realiza o tiene algún tipo de hobby que le permita desconectar del trabajo? (deporte, naturaleza, ocio…)
Do you have a suppport network that helps you process work-related stress? / ¿Cuenta con una red de apoyo que le ayude a afrontar el estrés relacionado con el trabajo?
What do you do after a difficult shift? / Después de un turno difícil, ¿qué hace?
How do you feel about self-care? Do you think that self-care can impact on how you care for others? / ¿Qué opina de cuidarse a sí mismo? ¿Cree que el cuidado personal puede influir en el cuidado de los demás?
What happens if you are overwhelmed by negative thoughts? How does your body and mind feel?/ ¿Qué ocurre si te abruman los pensamientos negativos?
Can you see your own faults and not judge them? / ¿Puedes ver los defectos y no juzgarlos?
**Block 4: What is Compassionate Care?**
How do you feel about the care you provide? Is it enough? /¿Cómo se siente respecto a los cuidados que realiza cada día?
Why do you think you feel that way? / ¿Por qué cree que se siente así XXXXXX ? (Tanto si la persona dice que se siente, triste, cansada, contenta…)
Can you give me any examples of care experiences that have made you feel this way XXXX? / ¿Ha tenido alguna experiencia con los cuidados prestados que le haya motivado a sentirse de esta manera XXXX?
From your overall experiences, both positive and negative, as a nurse providing care, could you explain what ingredients a nurse must possess in order to provide care appropriately? / Del conjunto de

experiencias, tanto positivas como negativas, como enfermera que presta cuidados, ¿me podría explicar que ingredientes o factores debe poseer una enfermera para prestar los cuidados de forma adecuada?
What is compassionate care in your opinión? / ¿Qué es el cuidado compasivo en tu opinión?
Can you give me an example of compassionate care from your practice? / ¿Puede darme un ejemplo de un acto de cuidado compasivo?
What does the concept of caring mean to you? / Para Ud. que significa el concepto de "cuidado"?
Do you think there is a difference between "caring" and "compassionate care"? Why? / Considera que hay alguna diferencia entre los conceptos "cuidado" o "cuidado compasivo?" ¿Por qué?
Here I will introduce a definition of the concept of compassionate care, so that the interviewer and interviewee can work from the same concept during the rest of the interview. Clarifying shared meanings throughout the interview will facilitate further analysis and ensure higher quality results (Kvale & Brinkmann 2009).
Compassionate care as: "a virtuous response that seeks to alleviate a person's suffering and needs through relational understanding and action" (Sinclair, McClement, Raffin-Bouchal, et al. 2016). Thus defined, compassionate qualities are essential to reduce patients' pain and suffering and promote their well-being (Halifax 2011). / Cuidado compasivo como: **"respuesta virtuosa que busca aliviar el sufrimiento y las necesidades de una persona a partir de una comprensión relacional y de la acción"** (Sinclair, McClement, Raffin-Bouchal, et al. 2016). Así definida, las cualidades compasivas son imprescindibles, para reducir el dolor y el sufrimiento de los pacientes y promover su bienestar (Halifax 2011).
With this definition in mind, do you feel that you have the opportunity to offer this type of care in practice? Can you give me an example? / Teniendo en cuenta esta definición, ¿cree que tiene la oportunidad de prestar este tipo de asistencia? ¿Puede darme un ejemplo?
How do you feel when a patient declines treatment or medication? / ¿Cómo se siente cuando un paciente rechaza el tratamiento o la medicación?
How do your personal values affect the care you give?<br>What are your core values? / ¿Cómo afectan sus valores personales a la prestación de cuidados?
Block 5: Proposals for Improvement…
What do you think would help the current nursing situation? That is, in the face of the global nursing shortage, high attrition rates and reported high levels of job stress, do you think there is a solution or measures we could take? / ¿Qué crees que ayudaría a la situación actual de la enfermería? Es decir, frente a la escasez mundial de enfermeras, las elevadas tasas de abandono y los altos niveles de estrés laboral reportados, ¿cree que hay una solución o medidas que podríamos adoptar?
What would you have liked to know when you started your student nursing career? / ¿Qué te gustaría haber sabido cuando empezaste a trabajar como enfermera?
Knowing what you do now, would you still choose to be a nurse? / Sabiendo lo que sabes ahora, ¿seguirías eligiendo la profesión de enfermera?

If you could return to the beginning of your studies, what advice would you give yourself? / Si pudiera volver al principio de su formación, ¿qué consejos se daría a sí mismo? (¿Qué le aconsejarías a una nueva enfermera en prácticas?)
Where you ever offered practical advice or training on how to deal with stress/grief/ los at university? Would it be useful? / ¿Le ofrecieron algún consejo práctico sobre cómo afrontar el estrés y la pérdida/duelo en la universidad? ¿Sería útil?
If you could add or change something on the nurse training programme, what would it be? / Si pudieras cambiar/ añadir algo de la formación de enfermería, ¿qué sería?
**To Conclude…**
Is there anything else you would like to talk about on the topics we have touched on, such as compassion, stress, burnout or your resources in the face of it? / ¿Hay algo más de lo que te gustaría hablar sobre los temas que hemos tocado, como la compasión, el estrés, el agotamiento o sus recursos frente ello?
Would you like to add anything else? / ¿Hay algo más que le gustaría añadir?
Thank you so much for your collaboration.

Appendix B

INFORMED CONSENT FORM ADULTS

Compassionate care; an exploration of nurses' inner resources in the face of burnout.

Name of participant:_______________________________

Objetive: The overall aim of this study is to gain detailed insight into how the internal resources used by nurses at different points in their careers affect their ability to provide compassionate care in high stress environments, with the ultimate goal of using the results to introduce improvements and tools into nursing education programs to improve mental health outcomes for students and future professionals.

Knowledge will be generated through grounded theory methodology, using semi-structured interviews with nursing students and nursing professionals, to contribute to the development of good practice and evidence-based health policy, by approaching nurses and addressing their current concerns, sensitivities and challenges.

Interviews will last approximately one hours and will be audio-recorded and then transcribed for in-depth analysis. All personal details will be excluded, and copies of the final draft will be provided should participants wish to read it. Participants will be able to withdraw at any time from the study. The recordings will be stored after transcription (for 5 years in the UIB repository) and only the written information will be used for analysis. The results will be anonymized so that they can be disseminated through academic articles and my doctoral thesis.

Researcher Contact Information:

Sarah-Louise d'Auvergne Flowers, Departamento de Enfermería y Fisioterapia - Universitat de les Illes Balears (s.flowers@uib.cat; Tfn.:00 44 971259976, ext. 1309)

Risks and Benefits of the project: This study has no risk or direct benefit to you, although your participation in this study will help pave the way toward better mental health management tools for nurses in the future. If you are distressed by the issues discussed, you may decline to answer any or all of the questions and, if you wish, you may terminate your participation at any time.

Recording and use of interviews: I agree to the (audio only) recording of the interview for research purposes, and I authorise the use of verbatim quotations from my interview, knowing that the conditions of confidentiality and anonymity shall be applied. I understand that the audio recording will be transcribed and then stored (5 years in the university confidential repository) and that only written data will be used. I also authorise the use of my transcribed interviews to be used for scientific dissemination purposes.

I UNDERSTAND THAT: my participation is voluntary and that I can withdraw at any time and without having to give explanations. Furthermore, I am aware that the confidentiality of my data is guaranteed under these terms: (1) data will be treated respecting confidentiality in accordance with current data protection regulations; (2) I am entitled to all the legal rights that are detailed and specified at the bottom of this consent form; (3) my data will only be used by the research team for scientific purposes and will never be transferred to third parties, except by legal obligation; furthermore, they will be kept for 5 years from the signature of this consent form; and (4) the legitimacy of the project is based on the collection of data by informed consent

(art. 6.1a of the General Data Protection Regulation, GDPR), and the processing of the data, as stated above, is the only way to fulfill the objectives of the research project (art. 6.1.e of the GDPR).

I DECALRE THAT: I have read the information on this document and I have been sufficiently informed about the study. In addition, I have been able to ask questions about the objectives and methodology applied in the project. Therefore:

1. I give my consent voluntarily and I know that I am free to withdraw from the study at any time, for any reason, without having to give explanations, without any negative repercussions to myself.

2. Finally, I agree to participate in the project and I have received a copy of this consent from.

Date (dd/mm/yyyy): _____________________

Participant	Lead Researcher

In compliance with the provisions of Organic Law 3/2018, of December 5, on the protection of personal data and the guarantee of digital rights, we inform you that the data collected will be included in one or more files managed by the UIB in the register of the processing activity authorized for the purpose, the purpose of which is to be able to carry out the ongoing investigation. The requested data are necessary to fulfill said purpose and, therefore, the fact of not obtaining them prevents it from being achieved. The UIB is responsible for the treatment of the data and, as such, guarantees the rights of access, rectification, opposition, deletion, portability, limitation of the treatment and not to be subject to automated individual decisions with respect to the data provided and treaties To exercise the rights indicated, you must write to: University of the Balearic Islands, General Secretariat, to the attention of the data protection officer, cra. de Valldemossa, km 7.5, 07122 Palma (Balearic Islands), or to the email address <dpo@uib.es>. You also have the right to complain to the control authority at: <https://www.aepd.es>. In the same way, the UIB undertakes to respect the confidentiality of its data and to use them in accordance with the purpose for which they were collected.
