# Supplementary material for: Compassionate Care: A Qualitative Exploration of Nurses’ Inner Resources in the Face of Burnout
Source: Nurs Rep. 2024 Jan 2;14(1):66–77. doi: 10.3390/nursrep14010006 (PMC10801579; doi:10.3390/nursrep14010006)
Supplement: Supplementary file 1 [file nursrep-14-00006-s001.zip › Figure S1 Recruitment poster.pdf]

Agotamiento, estrés, escasez de personal... ¿Te resulta familiar?

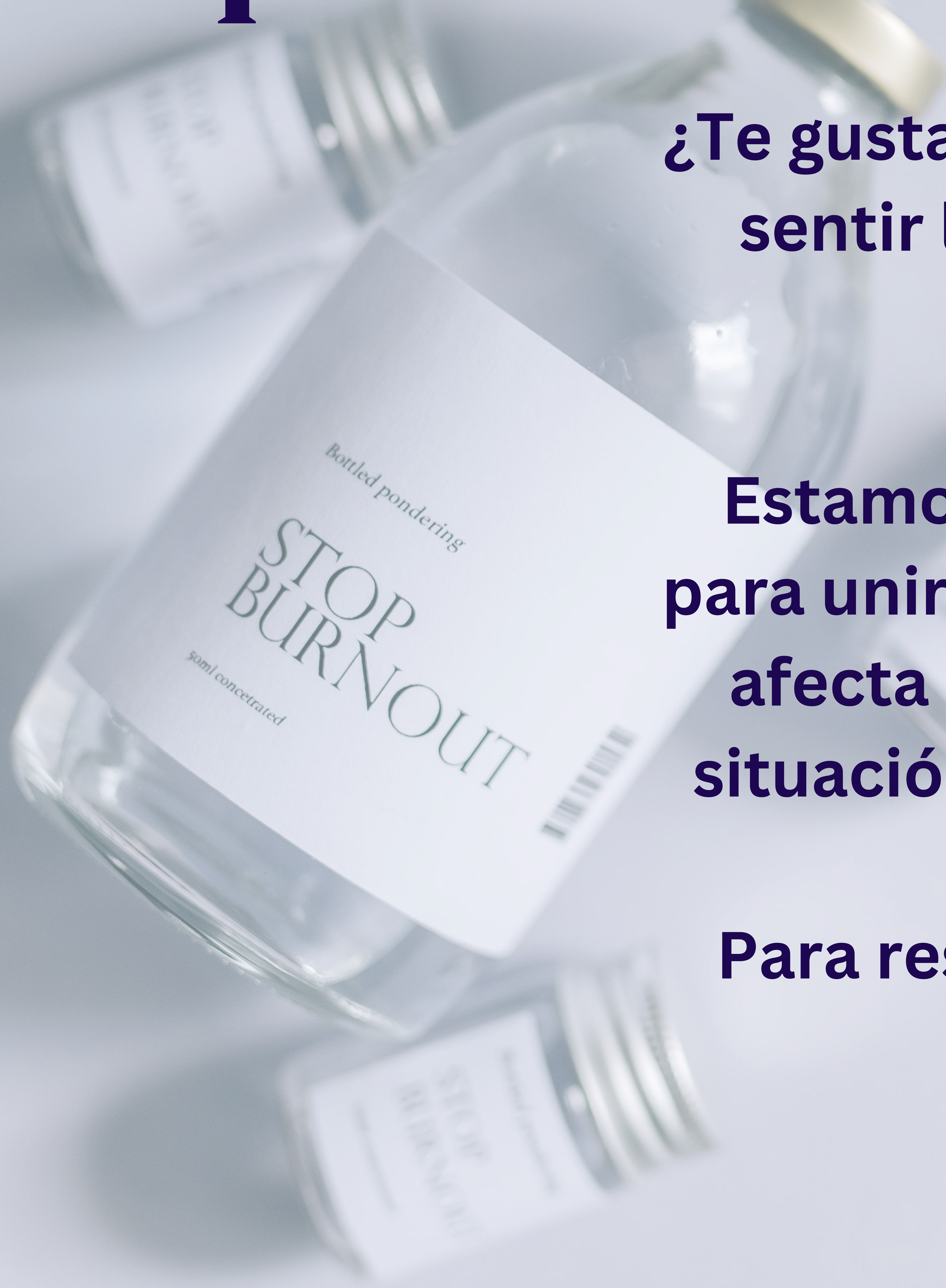

¿Te gustaría ser entrevistad@ sobre cómo te hace sentir la enfermería actual y cómo afecta a la atención al paciente?

Estamos buscando voluntari@s en enfermería para unirse a un estudio de la UIB sobre cómo nos afecta el burnout y como podemos mejorar la situación desde la perspectiva de la enfermería.

Para reservar tu entrevista o más información, ponte en contacto con:
s.flowers@uib.cat

Burnout, stress, short-staffing...
Sound familiar?

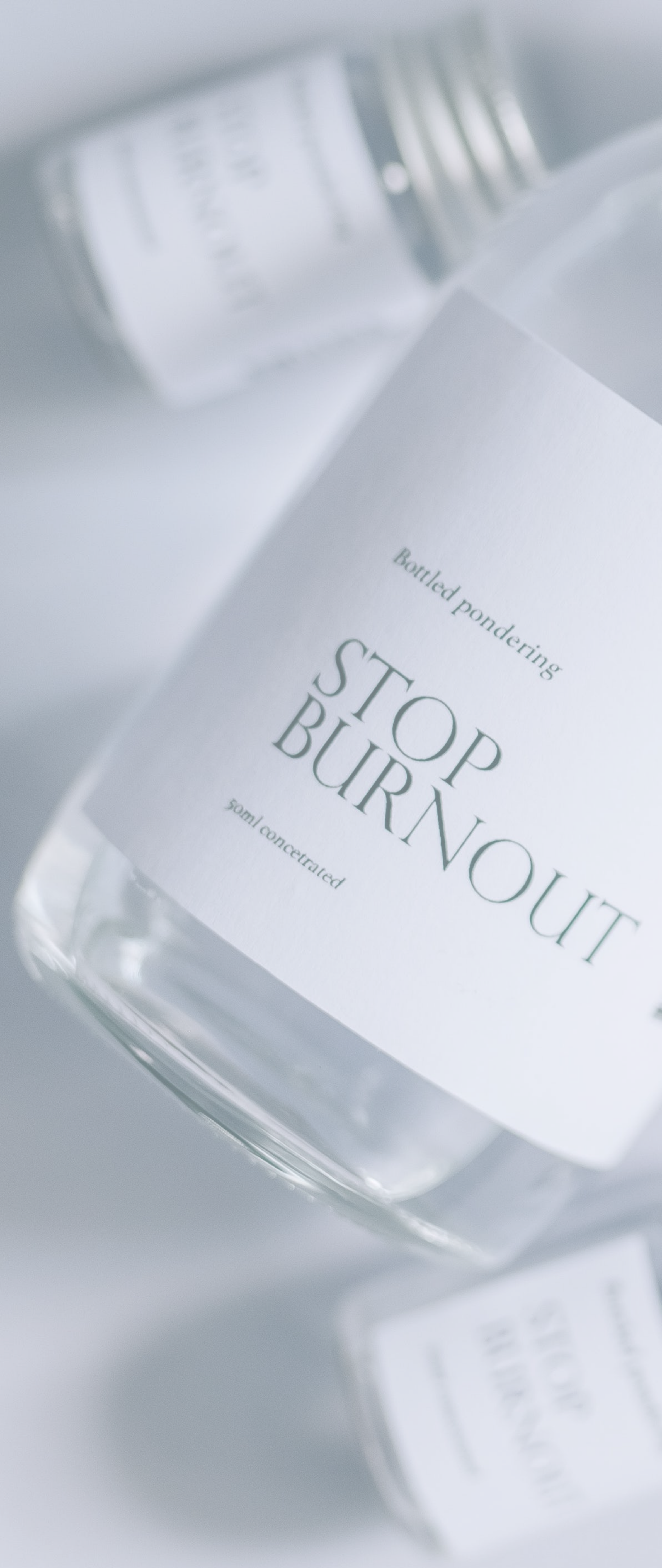

Would you like to be interviewed about how nursing today makes you feel and how it affects patient care?

We are looking for student & nurse volunteers to join a study at the UIB to explore how work conditions affect our capacity to deliver compassionate care, from the perspective of those working on the ground.

To reserve your interview or for more information, please contact:
s.flowers@uib.cat
